# Impact of the Surviving Sepsis Campaign clinical guideline of in sepsis mortality in a public health institution in Brazil

**DOI:** 10.1186/cc12905

**Published:** 2013-11-05

**Authors:** Suellen C de Aguiar, Guilherme F Garcia, Daniela N Ferreira, Francisco C de Souza, Valda MF Mendonça, Vanuza F Ribeiro, Lívia M Ferreira, Flávio D Capanema, Luana C de Carvalho, Marina F de Gomes

**Affiliations:** 1 Comissão Central de Protocolos Clínicos, Fundação Hospitalar do Estado de Minas Gerais, Belo Horizonte, MG, Brazil

## Background

Sepsis is the principal cause of mortality in intensive therapy units (ITUs) around the world [1]. Several international organizations created in 2002 the Surviving Sepsis Campaign (SSC), targeting the reduction of sepsis mortality in 25% during 5 years [2]. The Fundação Hospitalar do Estado de Minas Gerais (FHEMIG), Brazil, was incorporated in this campaign with eight hospitals (four general hospitals, one trauma hospital, one oncologic center, one infectious diseases center, one maternity hospital). The aim of this study is to evaluate the impact of using the SSC sepsis protocol in severe sepsis and sepsis shock lethality in the FHEMIG net hospitals.

## Materials and methods

This is a retrospective cohort study based on eight ITU public hospitals. The inclusion criteria were patients with severe sepsis and sepsis shock according to the SSC protocol, from January 2010 to December 2012, aged older than 18 years, which had a final outcome of hospital discharge or death. The sepsis lethality was compared annually from 2010 to 2012. Since 2010, the implementation of educative and managerial measures was based on the SSC guidelines: auditing of medical charts; education in sepsis care; issue of booklet and posters about sepsis; inclusion of sepsis information in the medical residence program; and collaboration of hospital directors in monitoring and giving information of the sepsis guideline. The study was approved by the Institutional Ethical and Research Committee. Data were collected and analyzed on EPIINFO software, using ANOVA test for comparisons with precision of 95%.

## Results

In the period of 3 years, 1,698 severe sepsis and sepsis shock patients were registered and 1,152 (67.84%) died. We verified a reduction of 12% (*P *= 0.0073) on lethality global. Hospitals 2 and 6 had a significant reduction on lethality, of 35% (*P *< 0.0001) and 17% (*P *= 0.0073) respectively (Table 1).

**Table 1:** Severe sepsis and sepsis shock death in the eight FHEMIG hospitals, from 2010 to 2012.

Hospital	2010			2011			2012			*P *value
		
	Number of patients	Death (*n*)	Death (%)	Number of patients	Death (*n*)	Death (%)	Number of patients	Death (*n*)	Death (%)	ANOVA
**1**	103	62	60.2	120	66	55	94	47	50	0.3578
**2**	59	52	88.1	114	87	76.3	110	63	57.3	0.0001
**3**	24	22	91.7	33	26	78.8	34	32	94.1	0.1292
**4**	68	57	83.8	71	63	88.7	68	54	79.4	0.3270
**5**	64	50	78.1	63	51	81	76	59	77.6	0.8821
**6**	94	75	79.8	92	54	58.7	115	76	66.1	0.0070
**7**	39	31	79.5	56	40	71.4	65	53	81.5	0.3954
**8**	49	15	30.6	38	10	26.3	49	7	14.3	0.1476

**Total**	**500**	**364**	**72.8**	**587**	**397**	**67.6**	**611**	**391**	**64.1**	**0.0074**

## Conclusions

The sepsis lethality is still high in this institution (64.1%), compared with the Public Hospitals in Brazil (59.6%) and the world rate (30.8%) [3]. After the adoption of managerial measures based on the SSC protocol, there was a significantly reduction in lethality, but only one hospital reached the target reduction of 25% on lethality. This heterogeneity could be explained by different engagements of the professional board and directory and different patient's profiles. The sepsis mortality is a major challenge in the world [4], and application of the SSC protocol led to a significant reduction in sepsis lethality.
